# Eat or Skip Breakfast? The Important Role of Breakfast Quality for Health-Related Quality of Life, Stress and Depression in Spanish Adolescents

**DOI:** 10.3390/ijerph15081781

**Published:** 2018-08-19

**Authors:** Rosario Ferrer-Cascales, Miriam Sánchez-SanSegundo, Nicolás Ruiz-Robledillo, Natalia Albaladejo-Blázquez, Ana Laguna-Pérez, Ana Zaragoza-Martí

**Affiliations:** 1 Department of Health Psychology, Faculty of Health Science, University of Alicante, 03690 Alicante, Spain; rosario.ferrer@ua.es (R.F.-C.); miriam.sanchez@ua.es (M.S.-S.); natalia.albaladejo@ua.es (N.A.-B.); 2 Department of Nursing, Faculty of Health Science, University of Alicante, 03690 Alicante, Spain; ana.laguna@ua.es (A.L.-P.); ana.zaragoza@ua.es (A.Z.-M.)

**Keywords:** breakfast, health-related quality of life, stress, depression, adolescents

## Abstract

This study examined the associations between eating or skipping breakfast and the quality of breakfast eaten on health-related quality of life (HRQOL), perceived stress and depression in 527 Spanish adolescents. Results showed differences in stress and two domains of HRQOL; Moods and Emotions and Parent Relations and Home Life between adolescent breakfast skippers and eaters, those having breakfast showing higher levels of stress and poor HRQOL. When breakfast quality was analyzed in breakfast eaters, adolescents who ate a good quality breakfast showed better HRQOL and lower levels of stress and depression than those who ate a poor or very poor quality breakfast. Further, breakfast skippers showed better HRQOL and lower levels of stress and depression than breakfast eaters who ate a poor or very poor quality breakfast. These findings indicate the importance of eating a good quality breakfast, rather than just having or not having breakfast. The conclusions of the present study are especially relevant for clinicians and nutritional educators, given the significant impact of breakfast quality on health-related quality of life, stress and depression observed in the adolescents studied.

## 1. Introduction

In Mediterranean countries, breakfast has been recognized as one the most important meal of the day [1]. Regular consumption of breakfast is associated with a range of benefits in children and adolescents including more adequate intakes of macro and micronutrients [2,3]; lower body mass index (BMI) [4]); higher cognitive performance [5,6]; and better levels of well-being [7] and quality of life [8]. Breakfast consumption among children and adolescents has shown to induce changes in metabolism, leading to improved diet quality and better food choices which may impact favorably on adolescents’ well-being and healthy habits throughout life, especially during early development [9,10]. Standard nutritional recommendations suggest that an ideal breakfast meal should contain 20 to 35% of daily energy derived from three food groups, including milk and milk derivatives, cereals (unrefined and whole grain) and fresh fruit or juice without added sugar [9].

Despite the potential physical and mental benefits of eating breakfast, several representative national surveys in North America and Europe have shown that breakfast skipping is highly prevalent [10,11] with an increased incidence from childhood through adolescence [12]. In the USA and Europe from 10 to 30% of young people report skipping breakfast and more than half show an inadequate/unbalanced breakfast pattern characterized by the intake of only one dairy food, one cereal food, or one piece of fruit [11,13]. Concretely in Europe, a recent study has shown that 24% of males and 33% of females are breakfast skippers [14]. However, other study conducted in ten European countries has demonstrated higher rates of breakfast skipping, exhibiting that 44% of females and 36% of males are breakfast skippers [15]. In addition to sex, other variables have shown to be moderators of breakfast consumption, such as the type of the day or the specific European country [16,17]. In this sense, it has been corroborated that prevalence of breakfast consumption was 74.4% for weekdays and 87.3% for weekends [16]. Regarding specific European countries, adolescents from Greece and Slovenia are the most breakfast skippers [17]. This fact demonstrates the need to analyze the phenomenon of breakfast consumption in each country deeply.

Breakfast skipping has been considered an important determinant of an unhealthy lifestyle that may serve as a proxy factor for other health-risk behaviors, including alcohol use, smoking, and sedentary lifestyle, as well as low educational attainment, mood changes, and depressive symptoms [18]. It has also been associated with a lower quality of life and chronic stress which may in turn increase the lifetime risk of cardiometabolic disease [19,20,21].

Different studies to date have provided data on breakfast habits among adolescents and their negative influence on psychological and physical status [14,15]. What is less clear, is whether the quality of breakfast has a significant impact on physical and mental health status in adolescents, rather than the factor of skipping or eating breakfast. Some recent findings examining the role of breakfast composition and quality of breakfast suggest that some specific foods, such as cereals or dairy products, might play a protective role in positive health benefits among adolescents, being associated with better nutrient intake, a higher level of physical activity and a reduced consumption of fats throughout the day [22,23]. Nevertheless, to our knowledge, extremely few studies have analyzed the relationship between quality of breakfast and mental health variables, such as depression, stress and HRQOL, in adolescents [16]. Furthermore, no studies have assessed whether skipping breakfast might be better than eating a poor quality breakfast. For these reasons, the present study sought to extend the existing literature on the relationship between breakfast quality and mental health in adolescents. Specifically, this study aimed to analyze the protective effects of eating breakfast on mental health and HRQOL. We hypothesized that a high quality breakfast, more than whether breakfast is eaten or skipped, would be positively related to all domains of HRQOL and to lower depressive symptoms and perceived stress in adolescents, indicating a possible protective effect of this dietary pattern. To test this hypothesis, we examined a sample of adolescent students living in a Mediterranean area in the east of Spain.

## 2. Materials and Methods

### 2.1. Procedure

The present study was a part of a large-scale study on Mediterranean diet and well-being conducted in schools in the Mediterranean city of Alicante, Spain. The study was approved by the Ethics Committee of the University of Alicante and by the management teams of the schools involved in the study (UA2015-1013). Participants included 527 high school students randomly selected from 5 public high schools in Alicante (Spain), of which 54.5% (*n* = 287) were female and 45.5% (*n* = 240) male. Participants ranged in age from 12 to 17 years, with a mean age of 14.30 (SD = 1.52). Inclusion criteria were: (1) Presence in the classroom on the day of the survey; and (2) ability to read and complete the questionnaires themselves. Prior to conducting the study, parents were asked to provide informed consent for their child to participate in the study. Students who were present on the day of data collection and assented to participate in the study were instructed to complete an anonymous online survey in the classroom. Participants were retained in the final sample only if they responded to all the questions involving the dependent variables assessing breakfast consumption and quality of life. Data were collected by a research assistant during the second and third trimester of the 2015–2016 academic year and sessions lasted approximately 60 min.

### 2.2. Measures

#### 2.2.1. Breakfast Eating and Breakfast Quality

In order to identify whether adolescents ate breakfast or not, and the quality of the breakfast they ate, we analyzed four items covering breakfast from the Mediterranean Diet Quality Index for children and teenagers (KIDMED) [24]. KIDMED is a questionnaire originally designed in Spain, and since employed in numerous international studies, for assessing adherence to the Mediterranean diet in children and young people. Consisting of 16 questions rated on a scale ranging from 0 to 12, this tool can be self-administered or administered by an interviewer following a standard protocol. To identify whether adolescents had breakfast, we considered scores on the following item: “Skip breakfast”, and to evaluate the quality of the breakfast eaten, we considered the responses to the following three items: (1) “Have cereal or other grain-based products (bread, toast, etc.) for breakfast”; (2) “Have some type of dairy product for breakfast”; and (3) “Have commercially baked goods (e.g., biscuits or pastries) for breakfast”. Based on the scores in these items, the sample was divided as follows: (1) Very poor quality breakfast: adolescents that did not eat bread/toast or cereal or dairy products for breakfast, but did eat commercially baked goods; (2) poor quality breakfast: adolescents that ate bread/toast/cereal and/or dairy products for breakfast but, at the same time, also ate commercially baked goods; and (3) good quality breakfast: adolescents that ate bread/toast/cereal and/or dairy products for breakfast and did not eat commercially baked goods.

#### 2.2.2. HRQOL—Health Related Quality of Life

KIDSCREEN-52 is a self-administered questionnaire measuring HRQOL in children and adolescents. It has 10 subscales covering the following dimensions: Physical Well-Being, Psychological Well-Being, Moods and Emotions, Self-Perception, Autonomy, Parent Relations and Home Life, Financial Resources, Social Support and Peers, School Environment and Social Acceptance. Items are scored on a five-point Likert-type scale. The Spanish version of KIDSCREEN-52 has shown to have good psychometric properties, with adequate validity, reliability, and cross-cultural comparability [25].

#### 2.2.3. Stress Perception

Stress perception was evaluated with the Perceived Stress Scale [26]. This questionnaire is composed of 4 items scored on a five-point Likert scale, ranging from “never” to “very often”. The total score is obtained by summing across all four items (maximum = 16). Higher scores are indicative of greater stress. The Spanish adaptation developed by Herrero and Meneses [27] employed in the present study has demonstrated adequate psychometric properties.

#### 2.2.4. Depression

The Short Web-Based version of the Center for Epidemiologic Studies Depression Scale (CES-D) [27,28], is a seven-item scale which evaluates the presence of depressive symptoms including the following dimensions: depressed affect, positive affect, somatic and retarded activity, and interpersonal problems. This instrument provides a general measure of depressive mood (with items including “I felt depressed” and “I was bothered by things that usually don’t bother me”). Responses are rated on a four-point Likert scale.

### 2.3. Data Analysis

*T*-tests were performed in order to assess differences between breakfast skippers and eaters in HRQOL, stress perception and depression. Multivariate analysis of variance (ANOVA) was employed to analyze differences in HRQOL factors between groups formed on the basis of breakfast quality (very poor quality, poor quality and good quality), and univariate ANOVA to identify differences between these groups in stress perception and depression. The same types of analyses (multivariate ANOVA for HRQOL and univariate ANOVA for perceived stress and depression) were employed to identify differences between breakfast skippers and eaters of very poor and poor quality breakfasts. Post-hoc analyses were implemented to examine specific differences between groups, with the Bonferroni adjustment for multiple comparisons. All statistical analyses were performed using IBM SPSS, Statistics for Windows, Version 24.0, considering any *p* < 0.05 as significant.

## 3. Results

### 3.1. Are There Differences between Breakfast Skippers and Eaters in HRQOL, Perceived Stress and Depression?

For HRQOL, differences between breakfast skippers and eaters were found in Moods and Emotion and Parent Relations and Home Life. Total HRQOL also differed between groups. In all cases, breakfast skippers had better HRQOL. Differences in perceived stress were also found, breakfast skippers showing lower levels of perceived stress than breakfast eaters. Regarding depression, differences between these groups were not significant (Table 1).

### 3.2. Are There Differences in HRQOL, Perceived Stress and Depression Depending on Breakfast Quality?

In order to evaluate differences within breakfast eaters by the quality of breakfast consumed, we investigated differences in HRQOL, perceived stress and depression between adolescents who ate very poor, poor and good quality breakfasts. In the case of HRQOL, significant differences were found in all of the dimensions evaluated: Physical Well-being, Psychological Well-being, Moods and Emotions, Self-Perception, Autonomy, Parent Relations and Home Life, Financial Resources, Social Support and Peers, School Environment, and Social Acceptance. There were also significant differences in total HRQOL. Post-hoc comparisons showed HRQOL was better in all dimensions for eaters of good quality breakfasts than eaters of very poor quality breakfasts (*p* < 0.001 in all cases). Similarly, eaters of good quality breakfasts obtained better scores in HRQOL dimensions than eaters of poor quality breakfasts (*p* < 0.001 in all cases), except in the case of Autonomy, Financial resources and Social acceptance. Furthermore, eaters of poor quality breakfasts obtained higher scores in Physical Well-being, Psychological Well-being, Moods and Emotions, Autonomy, Parent Relations and Home Life, Financial Resources, Social Support and Peers, School Environment, Social Acceptance and total HRQOL than eaters of very poor quality breakfasts (*p* < 0.05 in all cases) (Table 2).

With respect to perceived stress, significant differences were found between groups. Post-hoc analyses revealed that eaters of good quality breakfasts show lower perceived stress than eaters of very poor and poor quality breakfasts (*p* < 0.001). Likewise, eaters of poor quality breakfasts showed lower perceived stress than eaters of very poor quality breakfasts (*p* < 0.001). Finally, significant differences were also found in depression. Post-hoc comparisons found differences between all groups, eaters of good quality breakfasts showing lower levels of depression than eaters of poor or very poor quality breakfast (*p* < 0.001) and eaters of poor quality breakfasts less depression than eaters of very poor quality breakfasts (*p* < 0.001).

### 3.3. Is Skipping Breakfast Better Than Eating a Poor or Very Poor Quality Breakfast?

To investigate whether skipping breakfast is better than eating a poor or very poor quality breakfast, we analyzed differences in HRQOL, perceived stress and depression between adolescents in each of these groups. Regarding HRQOL, significant differences were found for all factors: Physical Well-being, Psychological Well-being, Moods and Emotions, Self-Perception, Autonomy, Parent Relations and Home Life, Financial Resources, Social Support and Peers, School Environment, and Social Acceptance, as well as total HRQOL. Post-hoc comparisons revealed that breakfast skippers obtained better results in all HRQOL dimensions than eaters of very poor quality breakfasts (*p* < 0.001 in all cases) and also better Physical Well-Being than eaters of poor quality breakfasts (*p* < 0.05).

In depression, significant differences were also found, breakfast skippers having lower levels of depression than eaters of very poor quality breakfasts (*p* < 0.001). Finally, regarding perceived stress, differences were again significant. Post-hoc analyses showed that breakfast skippers had lower perceived stress than eaters of very poor and poor quality breakfast (*p* < 0.05) (Table 3).

## 4. Discussion

This study examined the relationship between breakfast consumption and perceived stress, depressive symptoms and HRQOL among adolescent students. To our knowledge, this is the first study that demonstrates the importance of the quality of breakfast, for the maintenance of a good HRQOL and lower levels of stress and depression in adolescents. Contrary to previous studies, we found that compared to breakfast eating, skipping breakfast was significantly associated with better HRQOL and lower perceived stress. We investigated whether the quality of breakfast consumption rather than eating or not eating breakfast might help to explain these results. To test this hypothesis, we compared first (i) the differences in perceived stress, depressive symptoms and HRQOL between adolescents who reported consuming good, poor and very poor quality breakfasts, and second; (ii) differences in mental health and HRQOL between those who skipped breakfast and those who ate a poor or very poor quality breakfast.

Regarding the first set of comparisons, our findings suggest that a high-quality breakfast, characterized by consumption of cereal and dairy products, is associated with a better HRQOL and lower levels of perceived stress and depressive symptoms in adolescents. The positive contribution of breakfast to nutritional status and subjective health in children and adolescents has been previously linked to the quality of breakfast and the type of food consumed [29]. In children, a high quality breakfast has been defined as those including cereals, fruit or fruit juice and low-fat milk or other dairy products [9]. Cereals have commonly been considered a healthy breakfast food due to them containing a wide range of healthy micronutrients, protein, sugars, and carbohydrates and that they are frequently consumed with other healthy foods such as milk, which is known to be a significant source of calcium [18]. Previous studies have found that consumption of breakfast, in particular breakfast cereals, is associated with better physical and mental well-being and subjective health [30,31]. Our results are also consistent with the traditional view that eating a healthy breakfast is linked to lower mental distress, fewer depressive symptoms, positive mood and improved quality of life [32].

Although the mechanism through which breakfast contributes to reducing depression and stress remains unclear, several mechanisms have been proposed. Specifically, after eating breakfast, carbohydrates are converted into glucose producing changes in levels of acetylcholine, insulin, serotonin, glutamate and cortisol [23,33]. Ingestion of carbohydrates is particularly beneficial for the brain after night fasting as it reduces levels of cortisol production thereby decreasing the ‘stress’ signal. In addition, conversion of carbohydrates into glucose is essential for the formation of tryptophan, a precursor protein for the synthesis of serotonin, which regulates depressive symptoms, irritable mood and cognitive functioning [34,35]. Several studies support these assumptions, showing that individuals who eat a healthy balanced breakfast have a better mental health status, more positive attitudes to life [36], lower rates of depression [34] and high levels of quality of life than those who eat a poor quality breakfast [8]. Adolescents eating a high quality breakfast also display a healthy pattern of behavior including a relatively high level of physical activity and low consumption of fats throughout the day [22]. This positive effect of a good quality breakfast as a marker of a healthy lifestyle is particularly significant in childhood and adolescence, when dietary and other lifestyle pattern are initiated, resulting in long-term health and nutritional benefits in adulthood [8].

Our findings also suggest that adolescents with a regular habit of skipping breakfast have better HRQOL, less subjective stress and fewer depressive symptoms than those who eat a very poor quality breakfast, characterized by consumption of commercially baked goods. Although the lack of a standardized definition of what constitutes a poor quality breakfast, as well as different methods for measuring breakfast consumption, may lead to different results across studies examining the link between breakfast consumption and composition on health outcomes [37], previous studies in children and adults indicate that the consumption of high levels of added sugar, fat and commercially baked goods may contribute to dietary inadequacies that are not compensated for by other meals [38,39], and these might in turn affect HRQOL, depression and stress. Furthermore, a growing body of evidence suggests that there is a link between increased sugar consumption, major depression and oxidative stress in humans. Higher added sugar intakes have been reported in individuals at risk for depression [40] and neuroimaging studies confirm that exposure to pleasant tastes such as added sugar activate the same brain regions of the orbitofrontal cortex, anterior insula, and amygdala as those activated in patients with depression [41,42]. All of these factors could explain the results obtained in the present study, in which adolescents who consumed a poor or very poor quality breakfast showed poorer health outcomes than those who skipped breakfast. Some evidence suggest that calorie restriction of skipping breakfast may have more beneficial effects that a poor quality breakfast given that calorie restriction particularly, suppression of added sugar intakes has been shown to have metabolic benefits including neuroprotective, anti-aging and anti-inflammatory outcomes. Thus, it is possible that calorie restriction play a similar role in psychological wellbeing and quality of life. Nevertheless, futures studies should examine these hypotheses exhaustively and attempt to replicate this pattern of results [43].

Although the present study entails an important advance in the comprehension of the effects of breakfast on psychological functioning in adolescents, some limitations should be addressed. Firstly, the cross-sectional design of the study does not allow us to establish causal relationships between the variables studied. Longitudinal studies are needed to explore how breakfasts habits could play an important role on psychological wellbeing and quality of life. Secondly, dietary information from participants was based on self-report which may be subject to error, in particular, underreporting. Further, the quality of breakfasts was evaluated through three items of a previous validated questionnaire employed to evaluate adherence to the Mediterranean diet, rather than a specific instrument focused on assessing breakfast quality. Nevertheless, the relevant content of these items (intakes of cereals, dairy products and commercially baked goods) and the differences obtained between groups formed based on the scores on these items demonstrate their validity to stratify adolescents by the quality of breakfast they usually eat. Hence, the results obtained indicate the validity and usability of a short form to assess breakfast quality in this population, containing information about the intake of foods essential for what is traditionally considered a good-quality breakfast (namely, cereal and dairy products) [23]. Furthermore, the variables evaluated are key factors for improving our understanding of the important role that nutritional factors play in the psychological functioning and HRQOL of adolescents. However, several other potential outcomes that may be linked to quality of life and psychological wellbeing in adolescent student were not included in the current study. Future research should assess the impact of additional variables such as the cultural differences in breakfast, composition of breakfast, weight as well as the protective role of family in individuals who skipping breakfast. Despite these limitations, this study provides evidence of the important that breakfast play on health and psychological wellbeing.

## 5. Conclusions

To our knowledge, this is the first study that has shown the importance of the quality of breakfast, rather than just whether breakfast is eaten or skipped, for the achieving good HRQOL and low levels of stress and depression in adolescents. As previously described, breakfast can potentially affect mental health in several ways. Although most previous research has indicated that breakfast consumption is valuable for the mental and physical health of adolescents, few studies have gone a step further, analyzing the importance of the quality of the breakfast consumed. Our findings entail a significant advance in the field of nutrition education, in that they imply that nutritional programs should not only include strategies for promoting breakfast consumption, but should place emphasis on the eating of a healthy breakfast.

## Figures and Tables

**Table 1:** Differences between breakfast skippers and breakfast eaters in health-related quality of life (HRQOL), perceived stress and depression.

	Breakfast			
	Skippers	Eaters	Differences between Groups	Cohen’s d
	*n* = 140	*n* = 387		
HRQOL				
Physical Well-being	18.47 ± 3.43	17.90 ± 4.05	t(525) = 1.468, *p* = 0.143	0.11
Psychological Well-being	23.55 ± 4.70	23.25 ± 4.84	t(525) = 0.626, *p* = 0.532	0.05
Moods and Emotions	27.63 ± 5.72	26.49 ± 5.81	t(525) = 1.996, *p* = 0.046	0.17
Self-Perception	19.92 ± 3.49	19.33 ± 3.49	t(525) = 1.727, *p* = 0.085	0.15
Autonomy	19.38 ± 3.76	18.87 ± 4.39	t(285.426) = 1.311, *p* = 0.191	0.15
Parent Relations and Home Life	25.37 ± 4.55	24.41 ± 4.97	t(525) = 2, *p* = 0.046	0.17
Financial Resources	12.04 ± 2.69	11.85 ± 2.92	t(525) = 0.664, *p* = 0.507	0.05
Social Support and Peers	25.17 ± 3.72	25.01 ± 4.16	t(525) = 0.384, *p* = 0.701	0.03
School Environment	21.95 ± 4.51	21.19 ± 4.52	t(525) = 1.689, *p* = 0.092	0.14
Social Acceptance	13.30 ± 2.14	13.16 ± 2.26	t(525) = 0.623, *p* = 0.534	0.05
Total HRQOL	39.42 ± 4.73	38.19 ± 6.13	t(316.965) = 2.413, *p* = 0.016	0.27
Perceived stress	8.45 ± 2.38	9.11 ± 2.75	t(525) = −2.503, *p* = 0.013	0.21
Depression	17.52 ± 3.01	18.09 ± 3.34	t(525) = −1.748, *p* = 0.081	0.15

**Table 2:** Differences in HRQOL, perceived stress and depression between breakfast eaters depending on breakfast quality.

	Breakfast Eaters				
	*n* = 387				
Breakfast Quality	Very Poor	Poor	Good	Differences between Groups	η2partial
	*n* = 86	*n* = 86	*n* = 215		
HRQOL					
Physical Well-being	15.22 ± 4.16	16.95 ± 4.17	19.36 ± 3.22	F(2, 384) = 420.783, *p* = 0.0001	0.182
Psychological Well-being	19.23 ± 4.81	22.74 ± 4.66	25.06 ± 3.81	F(2, 384) = 580.633, *p* = 0.0001	0.234
Moods and Emotions	21.50 ± 5.57	26.17 ± 5.83	28.62 ± 4.52	F(2, 384) = 600.620, *p* = 0.0001	0.240
Self-Perception	17.95 ± 3.13	18.91 ± 3.46	20.05 ± 3.46	F(2, 384) = 120.537, *p* = 0.0001	0.061
Autonomy	15.51 ± 4.55	19.04 ± 4.22	20.15 ± 3.65	F(2, 384) = 410.454, *p* = 0.0001	0.178
Parent Relations and Home Life	20.03 ± 4.84	24.36 ± 5.13	26.18 ± 3.74	F(2, 384) = 610.496, *p* = 0.0001	0.243
Financial Resources	10.41 ± 3.29	11.98 ± 2.82	12.37 ± 2.61	F(2, 384) = 140.890, *p* = 0.0001	0.072
Social Support and Peers	22.45 ± 4.87	24.87 ± 4.46	26.10 ± 3.17	F(2, 384) = 260.780, *p* = 0.0001	0.122
School Environment	18.44 ± 4.47	20.56 ± 4.45	22.54 ± 4.01	F(2, 384) = 300.332, *p* = 0.0001	0.136
Social Acceptance	12.30 ± 2.79	13.23 ± 2	13.16 ± 2.26	F(2, 384) = 80.672, *p* = 0.0001	0.043
Total HRQOL	31.60 ± 5.53	37.75 ± 5.61	41 ± 4.21	F(2, 384) = 1150.234, *p* = 0.0001	0.375
Perceived stress	12.01 ± 2.37	9.24 ± 2.56	7.90 ± 1.98	F(2, 384) = 105.915, *p* = 0.0001	0.356
Depression	21.37 ± 2.40	18.41 ± 3.13	16.64 ± 2.74	F(2, 384) = 90.666, *p* = 0.0001	0.321

**Table 3:** Differences between breakfast skippers, eaters of very poor quality breakfasts and eaters of poor quality breakfasts in HRQOL, perceived stress and depression.

	Eaters of very Poor Quality Breakfasts	Eaters of Poor Quality Breakfasts	Breakfast Skippers	Differences between Groups	η2partial
	*n* = 86	*n* = 86	*n* = 140		
HRQOL					
Physical Well-being	15.22 ± 4.16	16.95 ± 4.17	18.47 ± 3.43	F(2, 309) = 190.081, *p* = 0.0001	0.110
Psychological Well-being	19.23 ± 4.81	22.74 ± 4.66	23.55 ± 4.70	F(2, 309) = 230.205, *p* = 0.0001	0.131
Moods and Emotions	21.50 ± 5.57	26.17 ± 5.83	27.63 ± 5.72	F(2, 309) = 310.444, *p* = 0.0001	0.169
Self-Perception	17.95 ± 3.13	18.91 ± 3.46	19.92 ± 3.49	F(2, 309) = 90.227, *p* = 0.0001	0.056
Autonomy	15.51 ± 4.55	19.04 ± 4.22	19.38 ± 3.76	F(2, 309) = 250.909, *p* = 0.0001	0.144
Parent Relations and Home Life	20.03 ± 4.84	24.36 ± 5.13	25.37 ± 4.55	F(2, 309) = 340.302, *p* = 0.0001	0.182
Financial Resources	10.41 ± 3.29	11.98 ± 2.82	12.04 ± 2.69	F(2, 309) = 90.485, *p* = 0.0001	0.058
Social Support and Peers	22.45 ± 4.87	24.87 ± 4.46	25.17 ± 3.72	F(2, 309) = 110.688, *p* = 0.0001	0.070
School Environment	18.44 ± 4.47	20.56 ± 4.45	21.95 ± 4.51	F(2, 309) = 160.276, *p* = 0.0001	0.095
Social Acceptance	12.30 ± 2.79	13.23 ± 2	13.30 ± 2.14	F(2, 309) = 50.531, *p* = 0.004	0.035
Total HRQOL	31.60 ± 5.53	37.75 ± 5.61	39.42 ± 4.73	F(2, 309) = 610.801, *p* = 0.0001	0.286
Perceived stress	12.01 ± 2.37	9.24 ± 2.56	8.45 ± 2.38	F(2, 309) = 58.585, *p* = 0.0001	0.275
Depression	21.37 ± 2.40	18.41 ± 3.13	17.52 ± 3.01	F(2, 309) = 48.204, *p* = 0.0001	0.238
